# Electric‐Field‐Driven Reversal of Ferromagnetism in (110)‐Oriented, Single Phase, Multiferroic Co‐Substituted BiFeO_3_ Thin Films

**DOI:** 10.1002/adma.202419580

**Published:** 2025-04-28

**Authors:** Takuma Itoh, Kei Shigematsu, Hena Das, Peter Meisenheimer, Kei Maeda, Koomok Lee, Mahir Manna, Surya Prakash Reddy, Sandhya Susarla, Paul Stevenson, Ramamoorthy Ramesh, Masaki Azuma

**Affiliations:** ^1^ Materials and Structures Laboratory Institute of Integrated Research Institute of Science Tokyo Yokohama 226‐8501 Japan; ^2^ Kanagawa Institute of Industrial Science and Technology Ebina 243‐0435 Japan; ^3^ Sumitomo Chemical Next‐Generation Eco‐Friendly Devices Collaborative Research Cluster Institute of Science Tokyo Yokohama 226‐8501 Japan; ^4^ Department of Materials Science and Engineering University of California Berkeley Berkeley CA 94720 USA; ^5^ Department of Physics Arizona State University Tempe AZ 85281 USA; ^6^ Materials Science and Engineering School for Engineering of Matter Transport and Energy Arizona State University Tempe AZ 85281 USA; ^7^ Department of Physics Northeastern University Boston MA 02115 USA; ^8^ Materials Sciences Division Lawrence Berkeley National Laboratory Berkeley CA 94720 USA; ^9^ Department of Physics University of California Berkeley Berkeley CA 94720 USA; ^10^ Department of Materials Science and Nanoengineering Department of Physics and Astronomy Rice University Houston TX 77251 USA; ^11^ Research Center for Autonomous System Materialogy Institute of Integrated Research Institute of Science Tokyo Yokohama 226‐8501 Japan; ^12^ Present address: Research Center for Magnetic and Spintronic Materials National Institute for Materials Science Tsukuba 305‐0047 Japan

**Keywords:** BiFeO_3_, multiferroic materials, magnetoelectric coupling, scanning probe microscopy, weak ferromagnetism

## Abstract

While multiferroic materials are attractive systems for the promise of ultra‐low‐power‐consumption computational technologies, electric‐field‐induced magnetization reversal is a key challenge for realizing devices at scale. Though significant research efforts have been working toward the realization of a material which couples ferroelectricity and ferromagnetism, there are few, even composite, systems which are practical for device scale applications at room temperature. Co‐substituted multiferroic BiFe_0.9_Co_0.1_O_3_ is a promising candidate system, due to coupled ferroelectricity and weak ferromagnetism at room temperature. Here, it is theoretically indicated that the ferroic orders in this material are statically coupled, where an in‐plane 109° ferroelectric switching event can result in the reversal of this out‐of‐plane component of magnetization, and the electric field‐induced magnetization reversal is experimentally observed. Such an in‐plane poling configuration is particularly desirable for device applications.

## Graphical abstract

Electric‐field‐induced magnetization reversal is a long‐sought‐after achievement in materials engineering for use in next‐generation, ultra‐low power consumption memory devices. This work demonstrates fully electric‐field‐driven switching of the out‐of‐plane component of magnetization in (110)‐oriented Co‐substituted BiFeO_3_ thin films at room temperature, enabled through careful engineering of thin film boundary conditions and crystallography.

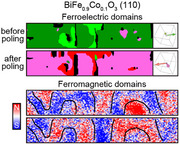

## 1 Introduction

The discovery and engineering of new multiferroic materials is highly technologically relevant due to the materials’ ability to couple magnetization to electric fields.^[^ ^1^, ^2^, ^3^, ^4^ ^]^ In particular, the possibility of developing a voltage‐write magnetic‐read‐out nonvolatile memory has attracted attention, as it could significantly lower the power consumption of electronic devices.^[^ ^5^, ^6^ ^]^ Conventional magnetic memory devices require an electric current to generate a magnetic field or spin current for reversing the magnetization, leading to significant losses due to Joule heating. Multiferroic materials with a strong magnetoelectric coupling, however, could eliminate the need for such an electric current. Though the search for a new multiferroic material has been an active area of research for years, there are still an exceedingly low number of room‐temperature, single‐phase multiferroic materials which show promise for device integration.^[^ ^7^, ^8^ ^]^ In order to build practical multiferroic devices, the key challenge is to design a material which demonstrates coupled ferroelectricity and ferromagnetism at room temperature, in a device‐integrable geometry.

BiFeO_3_ (BFO) is a magnetoelectric multiferroic material with both ferroelectric and antiferromagnetic orders coupled at room temperature.^[^ ^9^, ^10^, ^11^, ^12^, ^13^, ^14^ ^]^ This material has been extensively studied because of the coupling of these two ferroic orders through the Dzyaloshinskii‐Moriya interaction (DMI)^[^ ^15^, ^16^ ^]^ that is induced by the broken symmetries of the ferroelectric polarization and the rotation of the FeO_6_ octahedra.^[^ ^17^ ^]^ In bulk crystals, the spin‐5/2 state of the Fe^3+^ ion has a cycloidal modulation with a periodicity of 620 Å in 〈110〉_pc_ (“pc” denotes pseudo‐cubic) directions superimposed on the G‐type antiferromagnetic structure.^[^ ^10^, ^11^ ^]^ In the thin film limit, however, this magnetic structure can be engineered, facilitating the use of BFO in magnetoelectric devices. To this end, magnetic heterostructures have been explored to allow detection of the magnetic state through exchange coupling,^[^ ^4^, ^18^, ^19^, ^20^, ^21^ ^]^ but relegating this functionality to heterostructures of dissimilar materials introduces potential problems from the perspectives of processing and fatigue behavior.^[^ ^22^ ^]^

Previous results have shown that Co‐substituted BiFe_1−_ *_x_* Co*_x_* O_3_ shows both ferroelectricity and weak ferromagnetism at room temperature. Neutron powder diffraction on BiFe_0.8_Co_0.2_O_3_ revealed a spin structure change from the spin cycloid of intrinsic BFO at low temperatures to a collinear structure with spins perpendicular to the electric polarization above ≈120 K.^[^ ^23^ ^] 57^Fe Mössbauer spectroscopy on powder samples and (111)_pc_‐oriented thin films further confirmed the temperature‐induced spin structure change and indicated that this is a first order phase transition where the cycloidal and collinear phases coexist in the intermediate.^[^ ^24^, ^25^, ^26^ ^]^ Accordingly, canted weak ferromagnetism with a spontaneous magnetization of ≈0.06 μ_B_ perpendicular to the electric polarization was observed at room temperature in BiFe_0.9_Co_0.1_O_3_ (BFCO) thin films grown on SrTiO_3_ (STO) (111) substrates. The disappearance of the weak ferromagnetism at low temperatures eliminates the possibility of magnetic impurity as the origin of observed ferromagnetism.^[^ ^24^, ^26^ ^]^ Density functional theory (DFT) calculations and Monte‐Carlo simulations indicate that high‐spin Co^3+^ with a large spin‐orbit coupling induces a planar magnetic anisotropy and thus destabilizes the cycloidal modulation, experimentally confirmed by a synchrotron X‐ray emission spectroscopy.^[^ ^27^, ^28^ ^]^ These results manifest that this ferromagnetic moment comes from the disruption of the cycloidal modulation by partial substitution of Co for Fe. Importantly, a magnetization reversal accompanying polarization switching has been directly observed through a combination of piezoresponse force microscopy (PFM) and magnetic force microscopy (MFM) on (001)_pc_‐oriented BFCO thin films.^[^ ^29^, ^30^, ^31^ ^]^ This is in line with expectations from reports on BFO, where it has been shown that the complex ferroelectric switching leads to the rotation of the FeO_6_ octahedra that govern DMI, ultimately resulting in magnetoelectric determinism.^[^ ^4^, ^17^ ^]^ As Co‐substitution preserves the physical structure of BFO, both BFO and BFCO would be expected to follow a similar mechanism,^[^ ^32^, ^33^, ^34^ ^]^ where the direction of spontaneous magnetization can be correlated directly to the switching path. In the case of (001)_pc_‐oriented BFCO thin films, the reversal of the out‐of‐plane (OOP) component of the magnetization was detected after using an electric field to induce an OOP 71° switching event,^[^ ^29^, ^30^ ^]^ however, such a magnetization reversal was not observed under in‐plane (IP) 71° polarization switching.^[^ ^35^ ^]^ These results suggest that the presence or absence of a magnetization reversal depends on the manner and pathway of polarization switching.

From symmetry arguments, a 109° polarization switching would be expected to alter the DMI vector, and thus allow manipulation of the spontaneous magnetization of BFCO.^[^ ^36^, ^37^, ^38^ ^]^ However, previous works have shown that the 71° (180°) switching event is energetically preferred in (001)_pc_‐oriented ((111)_pc_‐oriented) thin films,^[^ ^36^ ^]^ making electrical reversal of the magnetization inaccessible. Herein, we focus on (110)_pc_‐oriented thin films to engineer the 109° ferroelectric switching event and access this electrical control of magnetization. We first theoretically demonstrate that this energetically unfavorable ferroelectric switching can be achieved through careful engineering of the thin‐film orientation and leads to the reversal of OOP magnetization. We experimentally confirm the magnetization reversal through the 109° switching using functional scanning probe microscopy in a specially designed configuration. Further this orientation, which is designed to allow for an OOP change in the magnetization when applying an IP electric field, is the most desirable architecture for a potential device.^[^ ^5^, ^6^, ^39^ ^]^

## 2 Results and Discussion

### 2.1 Theoretical Investigation of Magnetoelectric Switching Process

**Figure 1:**
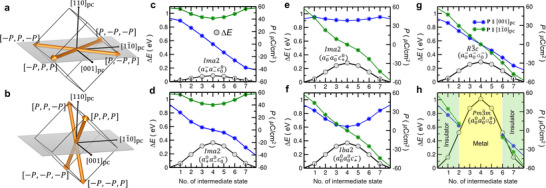
a,b) Fourfold degenerate polar states on the (110)_pc_ and ${\left(1\bar{1}0\right)}_{\mathrm{pc}}$ planes, respectively. c,d) Estimated energy barriers associated with the ferroelectric 71° switching paths that reverse the direction of polarization along the crystallographic [001]_pc_ axis (i.e., [*P*, −*P*, *P*] → [*P*, −*P*, −*P*]) from NEB calculations. e,f) Estimated energy barriers associated with the ferroelectric 109° switching paths that reverse the direction of polarization along the crystallographic ${\left[1\bar{1}0\right]}_{\mathrm{pc}}$ axis (i.e., [*P*, −*P*, *P*] → [−*P*, *P*, *P*]) from NEB calculations. g,h) Estimated energy barriers associated with the ferroelectric 180° switching paths (i.e., [*P*, −*P*, *P*] → [−*P*, *P*, −*P*]) from NEB calculations. The green and yellow regions denote the insulating and metallic states, respectively.

Numerous research studies have been conducted to control and engineer the properties of BFO thin films and hetero interfaces.^[^ ^4^, ^5^, ^11^, ^12^, ^38^, ^40^, ^41^, ^42^, ^43^, ^44^, ^45^, ^46^, ^47^, ^48^ ^]^ Compared to the parent system, BFCO and other transition metal‐substituted BFO^[^ ^27^, ^49^ ^]^ which exhibit a weak ferromagnetic state have received relatively less attention. Additionally, the relationship between the electric and magnetoelectric switching processes and the orientation of the BFCO thin film remains much less explored. Here, we theoretically investigate the possible polarization switching pathways and the accompanying magnetoelectric behavior in (110)_pc_‐oriented BFCO thin films and explore the effect of both compressive and tensile strains on the magnetoelectric properties. The origin of the ferroelectricity in BFCO lies in the polar distortion which transforms as the $\Gamma_{4}^{-}$ irreducible representation of the cubic $Pm\bar{3}m$ symmetry. The ground state *R*3*c* structure is characterized by octahedral tilts ($R_{4}^{+}$) and polar distortions ($\Gamma_{4}^{-}$). As both $R_{4}^{+}$ and $\Gamma_{4}^{-}$ are 3D, the corresponding order parameters, $Q_{R_{4}^{+}}$ and $Q_{\Gamma_{4}^{-}}$, are also 3D. The three components of $Q_{R_{4}^{+}}$, (θ, θ, θ), are the magnitude of anti‐phase FeO_6_ octahedral rotations around the pseudo‐cubic [100]_pc_, [010]_pc_, and [001]_pc_ axes, i.e., $a_{0}^{-}b_{0}^{0}b_{0}^{0}$, $a_{0}^{0}b_{0}^{-}a_{0}^{0}$, and $a_{0}^{0}a_{0}^{0}c_{0}^{-}$, respectively (here, in the Glazer notation, the superscript and subscript represent the octahedral out‐of‐phase rotation and electric polarization, respectively). Similarly, the three components of $Q_{\Gamma_{4}^{-}}$, (*P*, *P*, *P*), are the magnitude of the polar Bi‐displacements in the pseudo‐cubic [100]_pc_, [010]_pc_, and [001]_pc_ directions, i.e., $\;a_{+}^{0}b_{0}^{0}b_{0}^{0}$, $a_{0}^{0}b_{+}^{0}a_{0}^{0}$, and $a_{0}^{0}a_{0}^{0}c_{+}^{0}$, respectively. The combination of the octahedral rotations and Bi‐displacements leads to the formation of the eightfold degenerate polar states. Within the designed geometry of the BFCO films here, the eightfold degenerate polar states split into two sets of fourfold degenerate states, i.e., [(*P*, −*P*, *P*), (*P*, −*P*, −*P*), (−*P*, *P*, −*P*), (−*P*, *P*, *P*)] and [(*P*, *P*, *P*), (*P*, *P*, −*P*), (−*P*, −*P*, −*P*), (−*P*, −*P*, *P*)] as illustrated in **Figure** **1**a,b. Under the applied biaxial compressive strain in the (110)_pc_ plane, such as that from the STO (110) substrate, the latter set is higher in energy compared to the former. The opposite effect takes place under the application of tensile strain. As our focus is to investigate the possible routes of achieving OOP magnetization reversal by the application of IP electric field, using the nudged elastic band (NEB) method^[^ ^50^ ^]^ we systematically investigate the probable ferroelectric switching pathways within the crystallographic (110)_pc_ plane and estimate the energy barriers under the application of compressive strain, as shown in Figure 1. The 71° (i.e., [*P*, −*P*, *P*] → [*P*, −*P*, −*P*]) polarization switching event is associated with the lowest energy barrier of ≈88 meV (see Figure 1c), which is in agreement with the previous study^[^ ^4^ ^]^ and is mediated via the *Ima*2 phase having an $a_{+}^{-}a_{-}^{-}c_{0}^{0}$ octahedral rotation and electric polarization pattern. The energy barrier through the alternative *Ima*2 ($a_{+}^{0}a_{-}^{0}c_{0}^{-}$) 71°‐intermediate state is much higher, ≈365 meV (see Figure 1d). From these calculations, 109° (i.e., [*P*, −*P*, *P*] → [−*P*, *P*, *P*]) switching events are found to be three times higher in energy than 71° switching. The process through the *Ima*2 ($a_{0}^{-}a_{0}^{-}c_{+}^{0}$) intermediate involves surmounting a ≈281 meV energy barrier (see Figure 1e), and the *Iba*2 ($a_{0}^{0}a_{0}^{0}c_{+}^{-}$) intermediate is a ≈369 meV barrier (see Figure 1f). 180° polarization reversal (i.e., [*P*, −*P*, *P*] → [−*P*, *P*, −*P*]) is expected to occur via the $R\bar{3}c$ ($a_{0}^{-}a_{0}^{-}a_{0}^{-}$) non‐polar phase rather than the cubic $Pm\bar{3}m$ ($a_{0}^{0}a_{0}^{0}a_{0}^{0}$) metallic phase which is associated with an extremely high energy barrier, ≈1 eV (see Figure 1h), that is nearly three times higher compared to that associated with the former processes (see Figure 1g). The key point is that the IP 109° switching process has a ≈200 meV higher energy barrier than its 71° counterparts, implying it requires a higher electric field to activate. However, its potential is determined by the associated magnetization switching process, which we discuss next.

**Figure 2:**
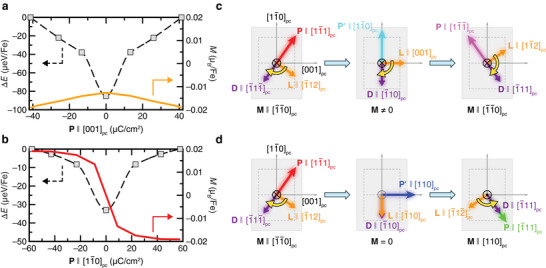
Magnetoelectric switching processes associated with the 71° and 109° polarization switching. a) In the case of 71° switching, the $Q_{\Gamma_{4}^{-}}$ order parameter rotates anti‐clockwise around [110]_pc_. The estimated relative energy of the anti‐clockwise rotation of G‐type antiferromagnetic order parameter **L** is presented here. b) In the case of 109° switching, the $Q_{\Gamma_{4}^{-}}$ order parameter rotates clockwise around [110]_pc_. The estimated relative energy of the clockwise rotation of G‐type antiferromagnetic order parameter **L** is presented here. c,d) Illustrated magnetoelectric switching processes mediated via *Ima*2 ($a_{+}^{0}a_{+}^{0}c_{0}^{-}$) and *Ima*2 ($a_{0}^{-}a_{0}^{-}c_{+}^{0}$) intermediate states corresponding to 71° and 109° polarization switching, respectively. For clarity, the component of DMI that leads to OOP magnetization is only illustrated.

Application of the IP electric fields along the [001]_pc_ and ${\left[1\bar{1}0\right]}_{\mathrm{pc}}$ directions is expected to change the direction of the polarization $\left(P\right)∥{\left[001\right]}_{\mathrm{pc}}$ and $P∥{\left[1\bar{1}0\right]}_{\mathrm{pc}}$ components of polarization, respectively. While the former process is expected to be mediated via a 71° anti‐clockwise rotation of **P** around [110]_pc_, the latter process involves a 109° clockwise rotation of **P** around [110]_pc_. To gain deeper insights into these processes, we conducted detailed investigations of the associated magnetoelectric phenomena and a comparative analysis of the 71° and 109° magnetoelectric switching processes. As summarized in **Figure** **2**, our results show that while the 71° anti‐clockwise rotation of $Q_{\Gamma_{4}^{-}}$ is not associated with the change in the direction of OOP magnetization (**M**), the 109° clockwise rotation of $Q_{\Gamma_{4}^{-}}$ leads to a 180° change in **M**. The OOP magnetization originates from the nearest‐neighbor anisotropic DMI between the magnetic ions which form G‐type antiferromagnetic order (**L**), i.e., M∝L×D, where both the component of DMI vector (**D**) that creates $M∥{\left[110\right]}_{\mathrm{pc}}$ (i.e., OOP magnetization) and **L** lie on the (110)_pc_ plane.^[^ ^15^, ^16^, ^27^ ^]^ Under the application of compressive strain, the magnetic states having an OOP component of the **L** vector were found to be higher in energy compared to $L⊥{\left[110\right]}_{\mathrm{pc}}$. To understand the magnetoelectric switching process, we analyze the initial, final, and intermediate states in detail, as illustrated in Figure 2. The intermediate states associated with the 71° and 109° switching processes have the same octahedral rotational pattern and space group symmetry *Ima*2. The $P∥{\left[1\bar{1}1\right]}_{\mathrm{pc}}\to P∥{\left[1\bar{1}\bar{1}\right]}_{\mathrm{pc}}$ rotation (71°) leads to anti‐clockwise rotation of **L** and **D**, i.e., $L∥{\left[\bar{1}12\right]}_{\mathrm{pc}}\to L∥{\left[001\right]}_{\mathrm{pc}}\to L∥{\left[1\bar{1}2\right]}_{\mathrm{pc}}$ and $D∥{\left[\bar{1}1\bar{1}\right]}_{\mathrm{pc}}\to D∥{\left[\bar{1}10\right]}_{\mathrm{pc}}\to D∥{\left[\bar{1}11\right]}_{\mathrm{pc}}$, respectively, hence the direction of **M** remains unaltered, as shown in Figure 2a,c. On the other hand, the $P∥{\left[1\bar{1}1\right]}_{\mathrm{pc}}\to P∥{\left[\bar{1}11\right]}_{\mathrm{pc}}$ rotation (109°) leads to clockwise and anti‐clockwise rotations of **L** and **D**, i.e., $\;L∥{\left[\bar{1}12\right]}_{\mathrm{pc}}\to L∥{\left[\bar{1}10\right]}_{\mathrm{pc}}\to L∥{\left[\bar{1}1\bar{2}\right]}_{\mathrm{pc}}$ and $D∥{\left[\bar{1}1\bar{1}\right]}_{\mathrm{pc}}\to D∥{\left[\bar{1}10\right]}_{\mathrm{pc}}\to D∥{\left[\bar{1}11\right]}_{\mathrm{pc}}$, respectively. Therefore, the 109° switching process leads to the reversal of OOP magnetization which is mediated via an intermediate state having *M*  =  0 as L∥D, as illustrated in Figure 2b,d. This phenomenon of magnetization reversal, however, does not accompany the 71° switching process which is associated with a much lower energy barrier and requires the application of a much lower switching voltage compared to the 109° switching process. The key point is to note that, in the (110)_pc_‐oriented BFCO thin films grown on a compressive strain exerting substrate, the direction of the OOP magnetization oriented along the [110]_pc_ axis can be reversed by applying an IP electric field along the ${\left[1\bar{1}0\right]}_{\mathrm{pc}}$ axis through the 109° polarization switching process.

### 2.2 Material Structure

**Figure 3:**
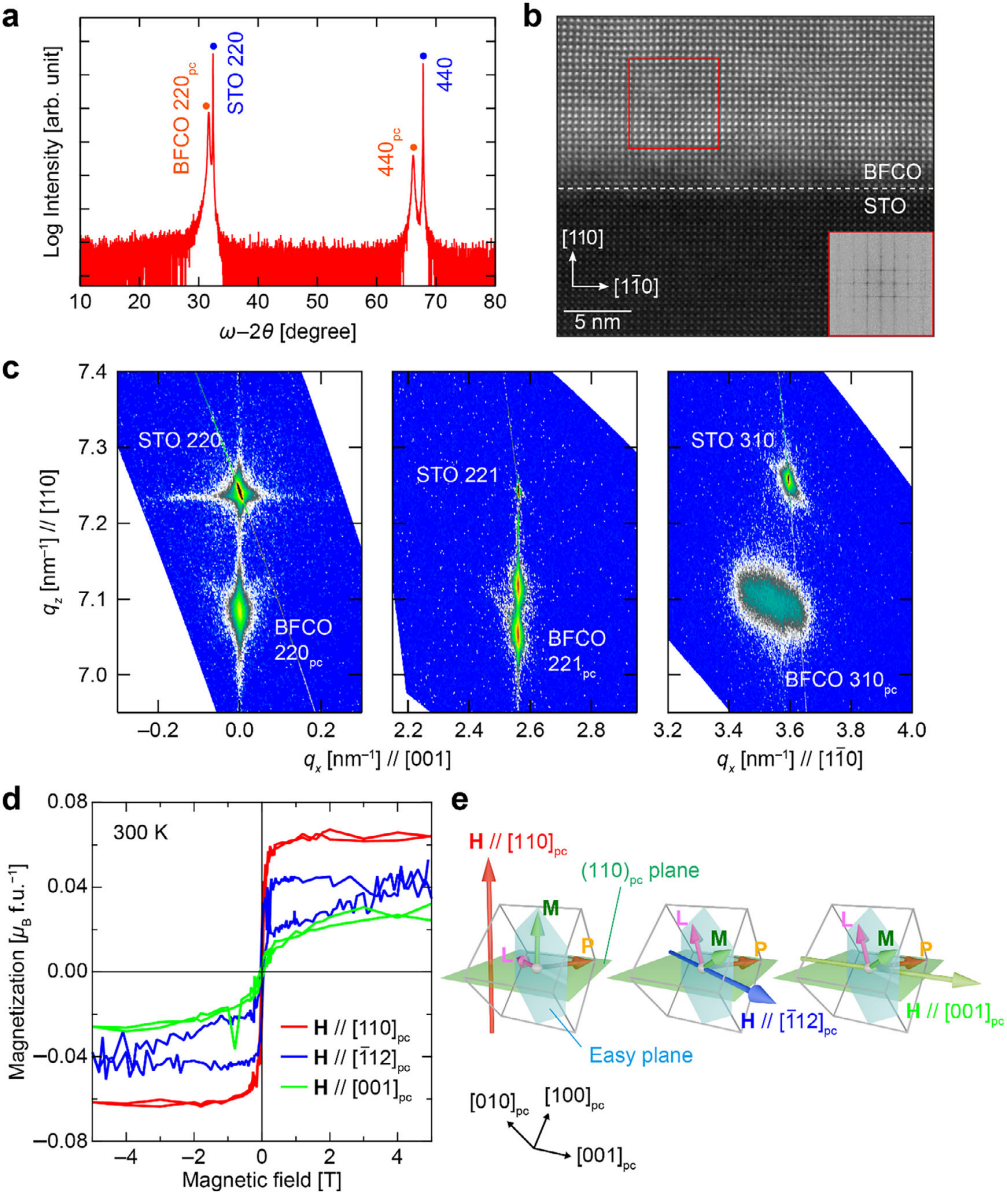
a) XRD *ω*−2*θ* pattern of BFCO/STO (110) thin film. b) Cross‐sectional HAADF STEM image of BFCO/STO (110) thin film, with the FFT inset of the red boxed region. c) XRD RSMs of BFCO/STO (110) around the 110, 221, and 310 reflections of STO. d) Magnetization hysteresis loops of BFCO/STO (110). Red line: along the OOP [110]_pc_ direction, blue line: along the IP ${\left[\bar{1}12\right]}_{\mathrm{pc}}$ direction, green line: along the IP [001]_pc_ direction. The relationships between the spin direction **L**, spontaneous magnetization **M**, and direction of the applied magnetic field **H** for polarization **P** in the ${\left[1\bar{1}1\right]}_{\mathrm{pc}}$ direction are also schematically shown in (e).

To verify the predicted magnetization reversal accompanning the 109° polarization switching, 60‐nm‐thick (110)_pc_‐oriented BFCO thin films are deposited on STO (110) substrates. **Figure** **3**a shows the X‐ray diffraction (XRD) *ω*−2*θ* pattern of films. Clear *hh*0 reflections from BFCO and STO indicate epitaxial growth of BFCO without impurities. The sharp interface between BFCO and STO is further ensured by the high‐angle annular darkfield (HAADF) scanning transmission electron microscopy (STEM) image shown in Figure 3b. The Fast Fourier Transform (FFT) pattern (Figure 3b, inset) confirms the single‐phase nature of the epitaxially grown BFCO on the [001]_pc_ zone axis. Reciprocal space maps (RSMs) around the 220, 221, and 310 reflections of the STO substrate are shown in Figure 3c. The splitting of the 221_pc_ reflection of BFCO into two segments indicates that a nearly rhombohedral structure is formed with polarization in the 〈111〉_pc_ directions.^[^ ^51^, ^52^, ^53^ ^]^ The 310_pc_ reflection exhibits a diffuse peak in *q*_x_ due to relaxation in the ${\left[1\bar{1}0\right]}_{\mathrm{pc}}$ direction.

The magnetic anisotropy of the as‐grown (110)_pc_‐oriented BFCO thin film is then evaluated through macroscopic measurements. Magnetization hysteresis loops along the OOP [110]_pc_, IP ${\left[\bar{1}12\right]}_{\mathrm{pc}}$, and IP [001]_pc_ directions are plotted in Figure 3d. Given that BFCO exhibits a magnetic easy plane perpendicular to the ${\left[1\bar{1}1\right]}_{\mathrm{pc}}$, representing one of the IP electric polarization directions, the [001]_pc_ direction should be the closest to the hard axis, ${\left[1\bar{1}1\right]}_{\mathrm{pc}}$, among the three measurement directions. Considering the spin direction along the six 〈121〉_pc_ directions and the spontaneous magnetization perpendicular to the spin direction,^[^ ^23^, ^29^ ^]^ the largest magnetization should appear along [110]_pc_. The experimentally observed saturation magnetizations are 0.06 *μ*_B_ f.u.^−1^ along [110]_pc_, 0.04 *μ*_B_ f.u.^−1^ along ${\left[\bar{1}12\right]}_{\mathrm{pc}}$, and 0.02 *μ*_B_ f.u.^−1^ along [001]_pc_, consistent with these expectations from symmetry. In addition, only the magnetization along [110]_pc_ saturates. This remnant magnetization of 0.04 *μ*_B_ f.u.^−1^ along [110]_pc_ agrees well with the value determined by diamond nitrogen vacancy (NV) center magnetometry discussed below. These results validate the proposed model and indicate the presence of a large OOP component of the spontaneous magnetization.

**Figure 4:**
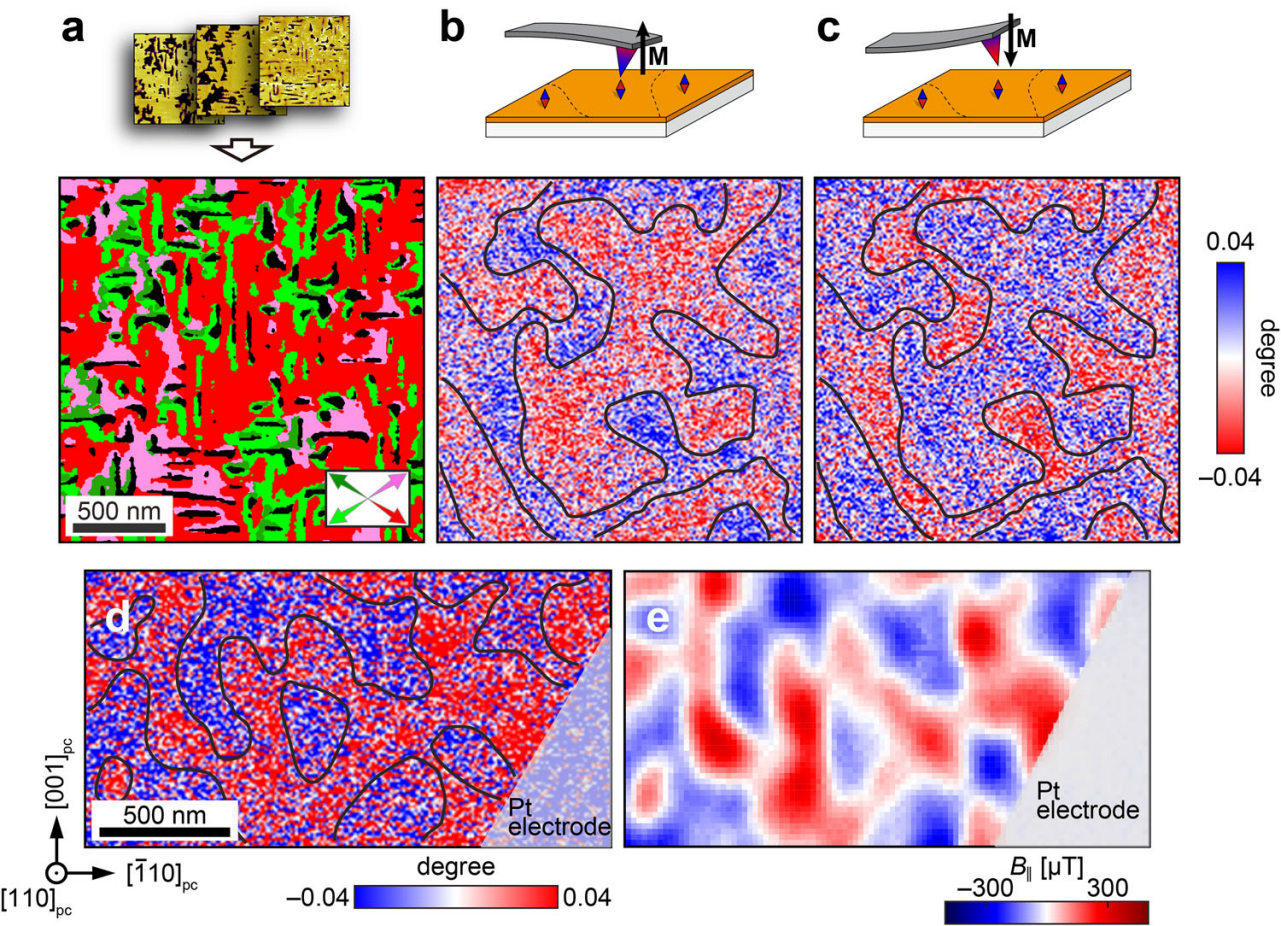
a) 3D‐PFM image constructed by superimposing three PFM phase images in Figure S1b (Supporting Information). The directions of the IP polarizations are indicated by the arrows of the same colors; the green, light green, red, and pink arrows represent the polarization directions of IP ${\left[1\bar{1}1\right]}_{\mathrm{pc}}$, IP ${\left[1\bar{11}\right]}_{\mathrm{pc}}$, IP ${\left[\bar{1}1\bar{1}\right]}_{\mathrm{pc}}$, and IP ${\left[\bar{1}11\right]}_{\mathrm{pc}}$, respectively. The black areas correspond the OOP polarization. b,c) MFM phase images of the same area as the 3D‐PFM image before and after the reversal of the magnetization of the cantilever. **M** is the magnetization vector of the tip. d) The MFM phase image at a different location from (a–c). e) Diamond NV center magnetometry image of the same area as Figure 4d. The black lines in the MFM images are guidelines showing the magnetic domain boundaries.

A 3D‐PFM image is constructed by superimposing three PFM phase images (Figure S1b, Supporting Information), shown in **Figure** **4**a. Among the eight possible polarizations along the 〈111〉_pc_ directions, the distribution of domains is dominated by the four variants fully contained within the (110)_pc_ plane. As the STO (110) substrate induces a large compressive strain, we assert that the OOP polarization in our as‐grown BFCO/STO (110) (black region) is downward.^[^ ^53^ ^]^ MFM of the same area is shown in Figure 4b. Due to the weak signal, in order to confirm that the observed contrast is magnetic in origin, the same location is imaged again after externally reversing the magnetization of the tip, noting a direct reversal of the signal (Figure 4c). The magnetic origin of the MFM image is confirmed by comparing the MFM image obtained at a different location (Figure 4d) with diamond NV center scanning magnetometry (Figure 4e). The two methods produce similar domain structures, despite sensing subtly different quantities (*B* vs *dB_z_*/*dz*).^[^ ^54^ ^]^ The film magnetization can then also be partially reconstructed from the NV magnetometry data by assuming that the magnetization lies along the OOP [110]_pc_ direction and using the experimental unit cell volume and film thickness (see Supporting Information). This approach provides a value of ≈0.04 *μ*_B_ f.u.^−1^ for the peak value of the domains, agreeing well with the perpendicular remanent magnetization measured from bulk magnetometry. The correlation between the ferroelectric and magnetic domains in the (110)_pc_‐oriented BFCO film, however, is less clear in comparison to (001)_pc_‐oriented BFCO thin films with a 71° boundary striped domain structure.^[^ ^31^, ^35^ ^]^ It has been reported for intrinsic BFO films that the cycloidal magnetic domain continues across the ferroelectric domain boundary and is not disrupted as obviously.^[^ ^55^ ^]^ Nevertheless, as the magnetic contrast of multiferroic domains in BFCO can be distinguished, the engineered anisotropy in these thin films can be investigated under magnetoelectric switching.

### 2.3 Magnetoelectric Behavior

**Figure 5:**
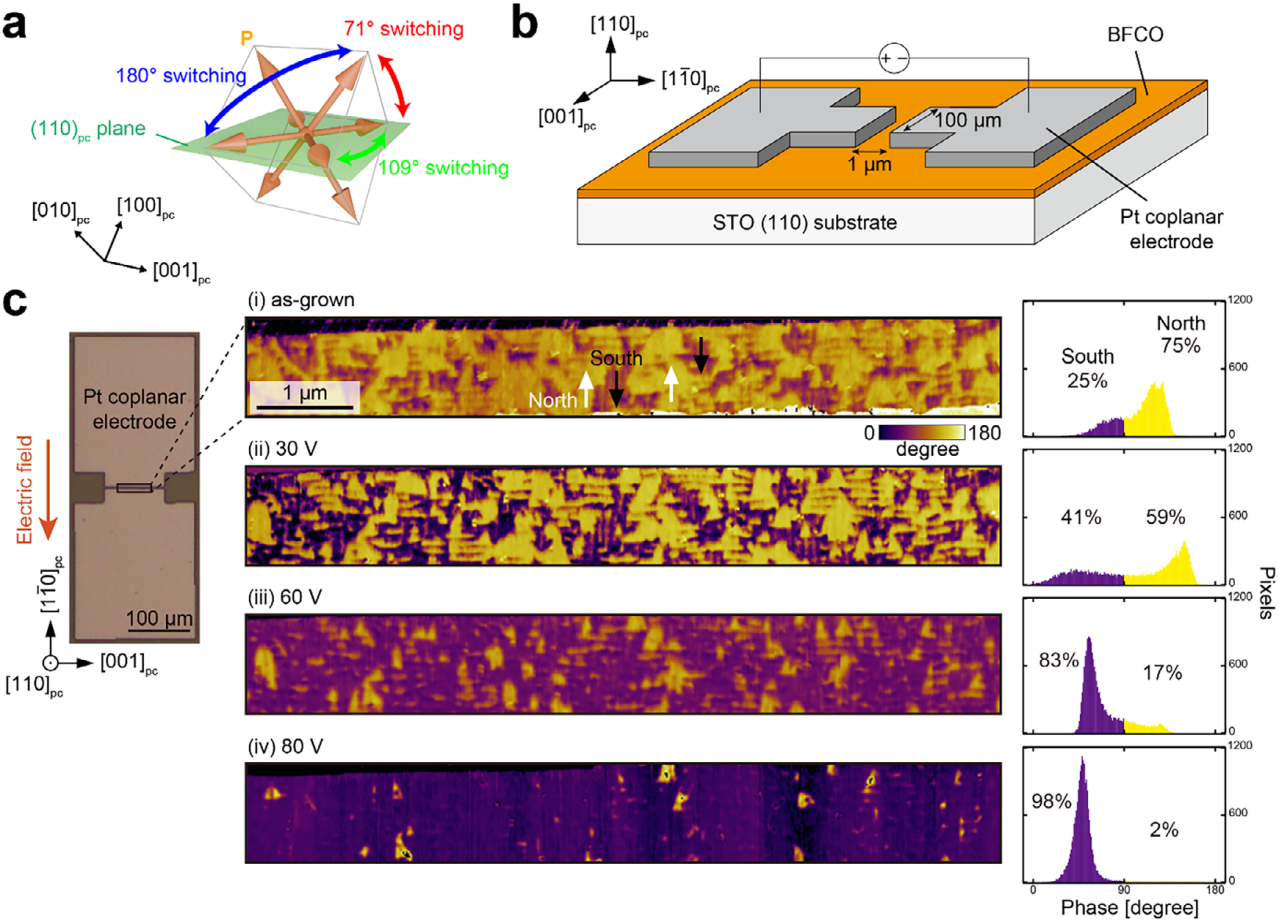
a) Eight possible 〈111〉_pc_ polarization directions and 71°, 109°, and 180° switching paths. b) Schematic illustration of Pt coplanar electrode deposited on BFCO for 109° IP polarization switching. c) Optical microscopy image of platinum electrodes and IP PFM phase images showing the polarization component (north or south) in the IP ${\left[\bar{1}10\right]}_{\mathrm{pc}}$ direction at various applied electric fields. The rightmost figures are the histograms of the PFM phase in the common region for each voltage.

In order to test electric‐field‐driven magnetization reversal in these samples, Pt coplanar electrodes were deposited on the (110)_pc_‐oriented BFCO thin film using typical photolithographic techniques, allowing an electric field to be applied in the IP ${\left[\bar{1}10\right]}_{\mathrm{pc}}$. This allows for IP 109° polarization reversal (**Figure** **5**a), where accompanying changes in the magnetic domain structure can be observed using MFM. A schematic illustration of the thin film and electrode configuration and an optical microscopy image of the test structure are shown in Figure 5b,c. The width of the electrodes was chosen to be ≈100 µm and the gap ≈1 µm to ensure spatial uniformity of the electric field. Figure 5c shows IP PFM phase images indicating the polarization component in the IP ${\left[\bar{1}10\right]}_{\mathrm{pc}}$ direction after applications of electric fields of different magnitudes. The gradual change of the up‐down domain fraction, quantified by the associated histograms, indicates that the polarization is gradually poled into the direction of the electric field. We observed that this IP switching was saturated after poling at 80 V (800 kV cm^−1^), where 98% of the polarization was oriented in the direction of the electric field. Although this value is much larger than the 22.5 V reported in previous work studying 71° switching,^[^ ^35^ ^]^ it is consistent with the theoretical prediction from Figure 1 that the energy barrier for 109° switching is about three times higher than that of 71° switching. Even at a strong electric field of 800 kV cm^−1^, the OOP components do not completely disappear, suggesting that electrically eliminating OOP polarization is extremely challenging. Reduction of the required electric field can potentially be achieved through optimization of the thin film fabrication process and device design, such as use of free‐standing film.^[^ ^33^ ^]^ Additionally, alternative switching mechanisms, such as mechanical or light‐induced methods, may be routes for ferroelectric switching with lower coercivity.

**Figure 6:**
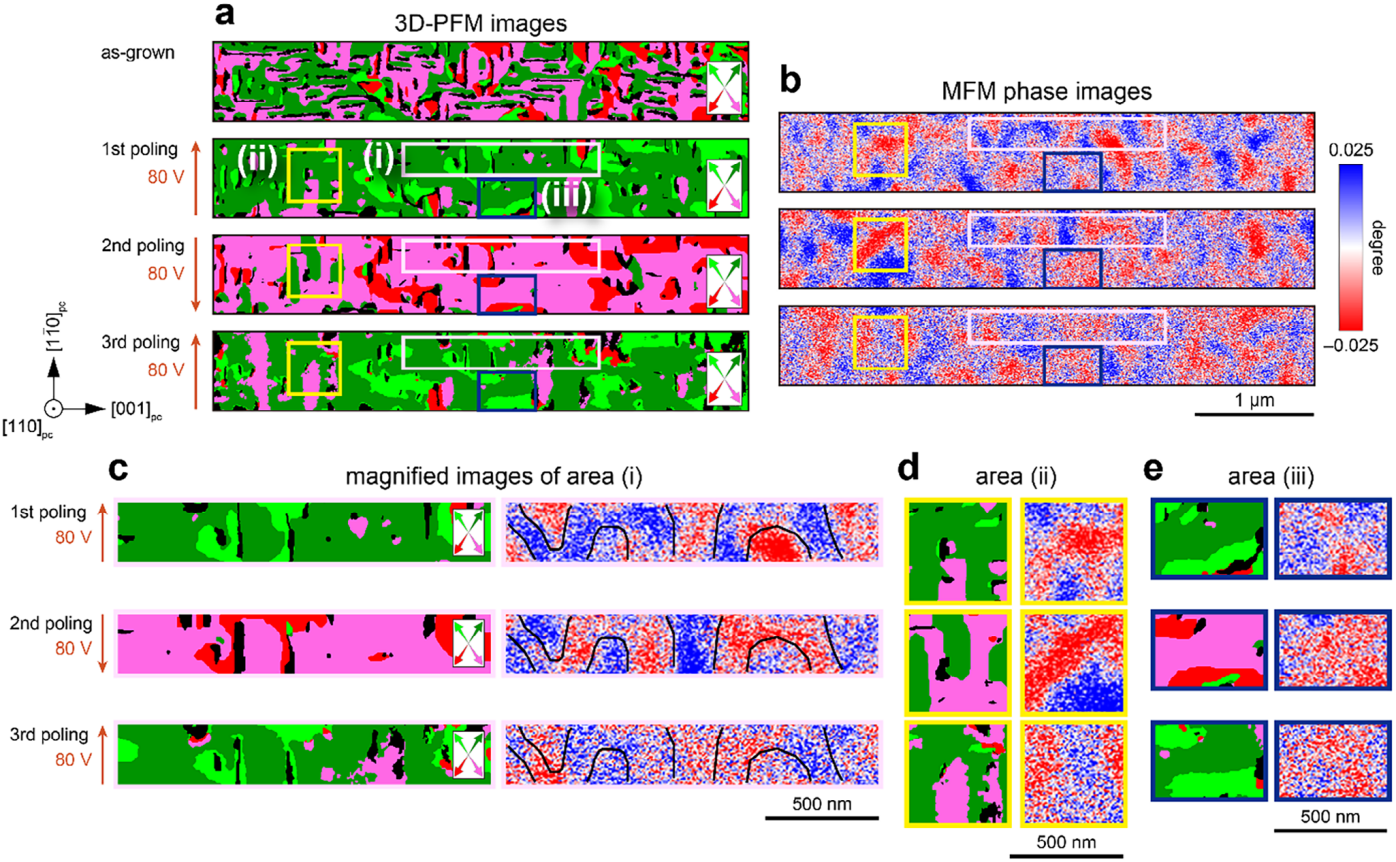
a,b) 3D‐PFM images and MFM phase images in the as‐grown state, after first, second, and third polings at 80 V via a Pt coplanar electrode. The polarization direction in each variant on the (110)_pc_ plane is indicated with an arrow of the same color on the right‐hand side of each 3D‐PFM image; the green, light green, red, and pink arrows represent the polarization direction of IP ${\left[1\bar{1}1\right]}_{\mathrm{pc}}$, IP ${\left[1\bar{11}\right]}_{\mathrm{pc}}$, IP ${\left[\bar{1}1\bar{1}\right]}_{\mathrm{pc}}$, and IP ${\left[\bar{1}11\right]}_{\mathrm{pc}}$, respectively. Black areas indicate polarization with an OOP component. c–e) 3D‐PFM images and MFM phase images of magnified images surrounded by the rectangles in (a,b). The black lines in the MFM images in Figure 6c are guidelines showing the magnetic domain boundaries.

Using these devices, the changes in the ferroelectric and magnetic domain structures after IP 109° polarization switching can then be investigated with another Pt electrodes on the same BFCO film. **Figure** **6**a,b shows vectorized 3D‐PFM and MFM images in the as‐grown state, after the first, second, and third applications of electric field. The raw PFM phase images are shown in Figure S3 and S4 (Supporting Information). The polarization direction of each domain variant in Figure 6a is indicated with an arrow of the same color on the right‐hand side of each 3D‐PFM image. The contrast changes from green (light green) to pink (red) or vice versa after each switching process; i.e., 109° polarization switching was achieved across the majority of the structure. After poling with the electric field, changes in the magnetic structure are observed from MFM measurements. Each successive ferroelectric switching induces magnetic domain change, indicating the presence of magnetoelectric coupling in the (110)_pc_‐oriented BFCO films even though the ferroelectric and magnetic domain structures do not have a clear correspondence. Figure 6a,b shows comparative PFM and MFM images of the switched structure, which are magnified in Figure 6c–e. In the magnified area c, ferroelectric domain boundaries primarily align parallel to the electric field. In areas which locally undergo 109° switching, the MFM contrast is reversed, demonstrating the first direct observation of magnetization reversal accompanying 109° polarization switching, consistent with the result of our theoretical investigation. To further support this observation, we conducted additional theoretical investigations based on the experimentally determined thin film lattice geometry. As observed in RSM around the 310_pc_ reflection, the strain in the ${\left[1\bar{1}0\right]}_{\mathrm{pc}}$ direction is relaxed, while the lattice parameter along the [001]_pc_ axis remains under ≈0.5% compressive strain. The key finding is that the qualitative nature of the magnetoelectric switching mechanism that accompanies 109° polarization switching remains unchanged, which drives the reversal of magnetization direction. Based on the detailed analysis (refer to Figures S5–S7 and the discussion in the Supporting Information), we conclude that structural relaxation does not introduce any qualitative differences in the reversal of electric polarization and magnetoelectric process compared to the theoretical predictions made by studying the system under 0.5% biaxial compressive strain.

This magnetization switching, however, is not clear in some regions. Figure 6d is the region where the 109° polarization switching is incomplete. To obtain further insights, we visualized how many switching events occurred in the measurement area shown in Figure S8 (Supporting Information). The region that underwent two 109° switchings accounted for 48%, while the one and zero 109° switchings were both 26%. Figure 6d corresponds to a zero switching area which is vertically elongated perpendicular to the interface of Pt‐electrode/BFCO. The formation of such a domain is potentially related to polarization fatigue at the planar‐type capacitor configuration.^[^ ^56^ ^]^ Indeed, a similar vertically elongated domain was reproduced when the number of polings was increased (Figure S9, Supporting Information). Given that BFCO demonstrates leakage currents more than one order of magnitude greater than those of BFO,^[^ ^57^ ^]^ and that a high voltage of 80 V was applied, forming incomplete switching regions due to polarization fatigue after only a few poling cycles is readily explicable. Figure 6e is the region where IP switching of 109° occurred, but lacked ferroelectric and ferromagnetic correlations even in the as‐grown state. The reason for this is not clear, but deduced from the difference of ferroelectric domain structures in Figure 6c, the presence of domain boundaries perpendicular to the electric field may be a significant influence which is removed upon cycling. The different nature of the correlation may stem from whether the ferroelectric domain has a 71° or 109° boundary. These suggest that, while the deterministic change in magnetization is locally true under particular switching events, the four‐variant domain structure of the films and the randomness with which the ferroelastic domain walls move prevent global determinism. The lower energy barrier for 71° polarization reversal compared to 109° polarization reversal, as discussed above, supports this result, indicating multiple polarization reversal paths rather than pure 109° switching may form a stochastic distribution. It is also possible that sequential OOP 71° polarization reversals result in 109° switching, but considering the even higher energy barrier for OOP 71° polarization reversal, the necessary electric field would destruct the domain structure. We thus hypothesize that deterministic magnetization reversal by electric field in this scheme could be achieved in carefully engineered domain structures, such as through the use of vicinally cut substrates^[^ ^52^, ^58^, ^59^ ^]^ or nanopatterned structures,^[^ ^60^ ^]^ where the ferroelectric domains are uniform.

## 3 Conclusion

In summary, we theoretically and experimentally demonstrate that careful selection of the crystallography and strain of BFCO thin films allows for the local control of OOP magnetization with an IP electric field. Functional scanning probe techniques were used to demonstrate the correlation of ferroelectric and magnetic domain structures after applying an electric field in the IP ${\left[1\bar{1}0\right]}_{\mathrm{pc}}$ direction to (110)_pc_‐oriented BFCO thin films. A reversal of the OOP magnetization was observed in areas which locally undergo 109° polarization switching, achieved by an electric field parallel to the domain boundary. This is the first evidence of magnetization reversal in BFCO thin films accompanying a 109° polarization reversal and is a milestone in the development of BFCO‐based ultra‐low power consumption electric‐field‐write and magnetic‐read‐out nonvolatile memory devices.

## 4 Experimental Section

### First‐Principles Calculations

To estimate the lowest energy path associated with the reversal of electric polarization under the application of an electric field, we used the NEB method.^[^ ^50^ ^]^ The first‐principles calculations were performed using the DFT + *U* method^[^ ^61^ ^]^ with the Perdew‐Burke‐Ernzerhof form of the generalized gradient approximation exchange‐correlation functional^[^ ^62^ ^]^ and using the projector augmented plane wave basis‐based method as implemented in the Vienna Ab initio Simulation Package (VASP).^[^ ^63^, ^64^ ^]^ We employed rotationally invariant approach of DFT + *U* method introduced by Dudarev et al.,^[^ ^65^ ^]^ which takes into account *U_eff_* =  *U* − *J*, where *U* and *J* correspond to the effective on‐site Coulomb and exchange parameters, respectively. As we conducted this study considering the high‐spin state of Co ions, we considered *U_eff_* |_*Fe*_ = 4.5 eV and *U_eff_* |_*Co*_ = 5.0 eV at the Fe and Co 3d states, respectively, as predicted in the previous study.^[^ ^27^ ^]^ The structural relaxations were performed by employing 0.001 eV Å^−1^ convergence criteria of the Hellmann‐Feynman forces. To mimic the experimental geometry, we constructed a supercell composed of 16 formula units of BiFe_1−x_Co_x_O_3_ compositions. Since no clear evidence of cation (Fe/Co) ordering was found either through theoretical calculations or experimental observations,^[^ ^27^ ^]^ we employed the methods outlined in references^[^ ^66^, ^67^ ^]^ to create the special quasi‐random structure of the BiFe_1−x_Co_x_O_3_ compositions. The technique involves closely replicating the perfectly random network for the initial few shells around a specific site, as implemented in the alloy theoretical automated toolkit.^[^ ^67^ ^]^ Within a 16‐formula unit cell size, we conducted NEB calculations by substituting two Fe ions with Co ions, resulting in a composition of BiFe_0.875_Co_0.125_O_3_, which closely resembles experimentally determined compositions and exhibits weak ferromagnetism.^[^ ^24^ ^]^ We used a 4 × 4 × 4 Monkhorst‐Pack Γ centered *k*‐points mesh and a kinetic energy cut‐off value of 520 eV. The NEB method involves the construction and optimization of intermediate configurations to determine the minimum energy transition path between the initial and the final states. We constructed seven such intermediate configurations between the initial and the final states. Along with the initial and final states, we calculated the electric polarization for each intermediate state using the Berry phase method^[^ ^68^ ^]^ as implemented in VASP. We calculated the magnetization originated from the anisotropic DMI between the magnetic ions by including spin‐orbit coupling self‐consistently as implemented in VASP. We also used the same method to estimate the value of DMI between the magnetic ions for the initial, intermediate, and final states of NEB.

### Sample Fabrication

Epitaxial 60‐nm‐thick BFCO thin films were deposited using pulsed laser deposition with a KrF excimer laser (λ = 248 nm) directly on (110)‐oriented STO single crystalline substrates. The STO substrates were ultrasonically cleaned in ethanol and acetone (sequentially) and were annealed at 1000 °C for 1 h in air before BFCO deposition. The substrate temperature and oxygen pressure during the BFCO deposition were maintained at 600–610 °C and 15 Pa. A sintered pellet of stoichiometric BFCO was ablated by the KrF excimer laser with a fluence and repetition frequency of 1.0 J cm^−2^ and 5 Hz, respectively. After the deposition, the films were cooled to room temperature for 40 min at an oxygen pressure of 5000 Pa to obtain a highly insulating property.^[^ ^31^ ^]^

### Material Characterization

The crystal structure of the BFCO thin film was investigated using XRD (Rigaku SmartLab) and STEM using a JEOL ARM 200F under the HAADF‐STEM mode operated at 200 kV and 30 mrad convergence angle. The magnetic measurements were conducted with a superconducting quantum interference device magnetometer (Quantum Design MPMS). Prior to observing the ferroelectric and ferromagnetic domains, 200‐nm‐thick Pt coplanar electrodes were patterned on the top of the film by using a conventional lift‐off process. The surface topography and the ferroelectric and magnetic domains were observed using contact‐mode atomic force microscopy, PFM, and MFM (Asylum Research Cypher S and MFP‐3D), respectively. ASYLEC.01‐R2 (Oxford Instruments) and MFMR (NANOWORLD) cantilevers were respectively selected for the PFM and MFM measurements. The consistent sample position was ensured by all surface voids being coincident within the measurement area. Any remaining misalignments are corrected by photo editing software, where the images were shifted and warped until they precisely match. All polarization directions along 〈111〉_pc_ were identified by making one OOP and two IP PFM measurements from which 3D‐PFM images were constructed.^[^ ^29^, ^30^, ^31^ ^]^ The magnetic origin of the MFM images was confirmed by observing the contrast reversal after the magnetization reversal of the cantilever and comparing the resulting MFM images with NV magnetometry. NV magnetometry data were measured on a Qnami ProteusQ system using a parabolic MX+ (100)‐oriented diamond cantilever. For magnetization reconstruction, the orientation and fly‐height on the NV center were determined using a reference CoFeB sample with known perpendicular magnetic anisotropy.^[^ ^69^ ^]^

## Conflict of Interest

The authors declare no conflict of interest.

## Supporting information



Supporting Information

## Data Availability

The data that support the findings of this study are available from the corresponding author upon reasonable request.
