# Supplementary material for: Electric‐Field‐Driven Reversal of Ferromagnetism in (110)‐Oriented, Single Phase, Multiferroic Co‐Substituted BiFeO3 Thin Films
Source: Adv Mater. 2025 Apr 28;37(29):2419580. doi: 10.1002/adma.202419580 (PMC12288817; doi:10.1002/adma.202419580)
Supplement: Supplementary file 1 — Supporting Information [file ADMA-37-2419580-s001.pdf]

ADVANCED MATERIALS

Supporting Information

for *Adv. Mater.*, DOI 10.1002/adma.202419580

Electric-Field-Driven Reversal of Ferromagnetism in (110)-Oriented, Single Phase, Multiferroic Co-Substituted $BiFeO_3$ Thin Films

Takuma Itoh, Kei Shigematsu*, Hena Das*, Peter Meisenheimer, Kei Maeda, Koomok Lee, Mahir Manna, Surya Prakash Reddy, Sandhya Susarla, Paul Stevenson, Ramamoorthy Ramesh and Masaki Azuma*

Supplementary Information

Electric-field-driven reversal of ferromagnetism in (110)-oriented, single phase, multiferroic Co-substituted $BiFeO_3$ thin films

Takuma Itoh, Kei Shigematsu*, Hena Das*, Peter Meisenheimer, Kei Maeda, Koomok Lee, Mahir Manna, Surya Prakash Reddy, Sandhya Susarla, Paul Stevenson, Ramamoorthy Ramesh, Masaki Azuma*

T. Itoh[†], K. Shigematsu, H. Das, K. Maeda, K. Lee, M. Azuma
Materials and Structures Laboratory, Institute of Integrated Research, Institute of Science Tokyo, Yokohama 226-8501, Japan
E-mail: ITOH.Takuma@nims.go.jp, kshigematsu@msl.iir.isct.ac.jp, hdas@msl.iir.isct.ac.jp

K. Shigematsu, H. Das, M. Azuma
Kanagawa Institute of Industrial Science and Technology, Ebina 243-0435, Japan

K. Shigematsu, M. Azuma
Sumitomo Chemical Next-Generation Eco-Friendly Devices Collaborative Research Cluster, Institute of Science Tokyo, Yokohama 226-8501, Japan

P. Meisenheimer, R. Ramesh
Department of Materials Science and Engineering, University of California Berkeley, Berkeley, California 94720, USA

M. Manna
Department of Physics, Arizona State University, Tempe, Arizona 85281, USA

S. P. Reddy, S. Susarla
Materials Science and Engineering, School for Engineering of Matter, Transport and Energy, Arizona State University, Tempe, Arizona 85281, USA

P. Stevenson
Department of Physics, Northeastern University, Boston, Massachusetts 02115, USA

R. Ramesh
Materials Sciences Division, Lawrence Berkeley National Laboratory, Berkeley, California 94720, USA

R. Ramesh
Department of Physics, University of California Berkeley, Berkeley, California 94720, USA

R. Ramesh
Department of Materials Science and Nanoengineering, Department of Physics and Astronomy, Rice University, Houston, Texas 77251, USA

M. Azuma
Research Center for Autonomous System Materialogy, Institute of Integrated Research, Institute of Science Tokyo, Yokohama 226-8501, Japan

[†]Present address: Research Center for Magnetic and Spintronic Materials, National Institute for Materials Science, Tsukuba 305-0047, Japan

Surface topography and ferroelectric domains of an as-grown $(110)_{pc}$-oriented $BiFe_{0.9}Co_{0.1}O_3$ (BFCO) thin film

Topography of the obtained film measured via atomic force microscopy shows an root mean square of surface roughness less than 0.3 nm, which is sufficiently flat for the piezoresponse force microscopy (PFM) and magnetic force microscopy (MFM) measurements (**Figure S1**a). In-plane (IP) horizontal, IP vertical, and out-of-plane (OOP) PFM images of the same area are shown in Figure S1b. Purple areas in the OOP phase image are assigned to the OOP polarization variants due to the locally larger PFM amplitude. A 3D-PFM image constructed by superimposing these three PFM phase images is shown in Figure 4a.

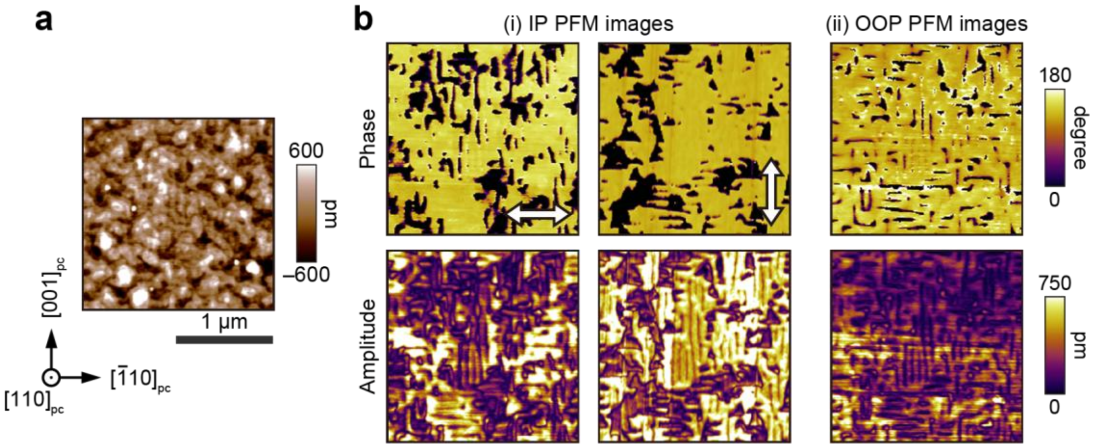

Figure S1 | a) An atomic force microscopy image of BFCO/$SrTiO_3$ (110) thin film. b) (i) IP PFM images identifying the polarization component in the IP $[\bar{1}10]_{pc}$ direction (left) and IP $[001]_{pc}$ direction (right) in the as-grown state. The arrows at the bottom right indicate the detection directions of the electric polarizations. (ii) OOP PFM image showing the polarization component in the OOP $[110]_{pc}$ direction.

Diamond nitrogen vacancy (NV) center magnetometry

Due to the linear relationship of magnetic field components in Fourier space,[1,2] the B_x, B_y, and B_z magnetic field components above the sample surface can be reconstructed from a single NV measurement. By calibrating the height of the NV defect above the sample surface using a perpendicularly magnetized structure, as described in Ref. 2 and 3, and assuming an Ising-like uniaxial anisotropy of the magnetization in the sample, the possible surface magnetization of the sample can be constrained to allow for back-calculation of the magnetization distribution. Using the procedure outlined in Ref. 1, we solve for the sample magnetization assuming possible uniaxial anisotropies along the sample $[110]_{pc}$ (OOP), $[\bar{1}01]_{pc}$, $[001]_{pc}$ (IP), and $[\bar{1}10]_{pc}$ (IP).

Defined in Ref 1 the relationship between magnetization and magnetic field above a surface can be expressed in Fourier space as:

$$\mathbf{b} = \begin{bmatrix} b_x \\ b_y \\ b_z \end{bmatrix} = -\frac{1}{\alpha} \begin{bmatrix} k_x^2 & \frac{k_x k_y}{k} & \mathrm{i}k_x \\ \frac{k_x k_y}{k} & k_y^2 & \mathrm{i}k_y \\ \mathrm{i}k_x & \mathrm{i}k_y & -k \end{bmatrix} \begin{bmatrix} m_x \\ m_y \\ m_z \end{bmatrix} = -\frac{1}{\alpha}\mathbf{Dm}, \quad (1)$$

$$\alpha = \frac{2\mathrm{e}^{kz}}{\mu_0},$$

Where $\mathbf{b}(k_x, k_y, z)$ and $\mathbf{m}(k_x, k_y)$ are the 2D Fourier transforms of the magnetic field and surface magnetization density respectively, k_i is a reciprocal space vector, $k = \sqrt{k_x^2 + k_y^2}$, and z is the observation height above the surface. Because of the linear dependency between $B_{x,y,z}$, B_i can be reconstructed by first solving for B_z using

$$m_z = \frac{\alpha b_z}{k} = -\frac{\alpha b_x}{\mathrm{i}k_x} = -\frac{\alpha b_y}{\mathrm{i}k_y}$$

and

$$m_z = \frac{-\alpha b_{\theta,\phi}}{u_x \mathrm{i}k_x + u_y \mathrm{i}k_y - u_z k}$$

where the rotation vector $\mathbf{u} = [u_x, u_y, u_z] = [\sin\theta\cos\phi, \sin\theta\sin\phi, \cos\theta]$, $\theta \approx 54°$ and ϕ are the spherical angles of the axis of the NV center, and $b_{\theta,\phi}$ is the Fourier transform of the measured NV data. Thus, to calculate b_i from $b_{\theta,\phi}$,

$$b_z = \frac{b_{\theta,\phi}}{-u_x \mathrm{i}k_x/k + -u_y \mathrm{i}k_y/k + u_z},$$
$$b_x = -\frac{\mathrm{i}k_x b_z}{k},$$
$$b_y = \frac{\mathrm{i}k_y b_z}{k}.$$

Because the inverse of Eq. 1 is not unique, to then calculate $\mathbf{m}$ directly from b_i using Eq. 1 it must be further constrained, in this case assuming an Ising spin configuration along an axis $\mathbf{l}$, such that

$$\begin{bmatrix} \cdots \\ \cdots \\ b_z(k_x, k_y, z) \end{bmatrix} = -\frac{1}{\alpha}\begin{bmatrix} \cdots & \cdots & \cdots \\ \cdots & \cdots & \cdots. \\ \mathrm{i}k_x & \mathrm{i}k_y & -k \end{bmatrix}\begin{bmatrix} l_x \\ l_y \\ l_z \end{bmatrix} m_l(k_x, k_y),$$
$$b_z = -\frac{1}{\alpha}(\mathrm{i}k_x l_x + \mathrm{i}k_y l_y - k l_z) m_l,$$

where $\mathbf{l}$ is a unit vector in the lab frame and $m_l(k_x, k_y)$ is the Fourier transform of the magnetization density pointing along $\mathbf{l}$.

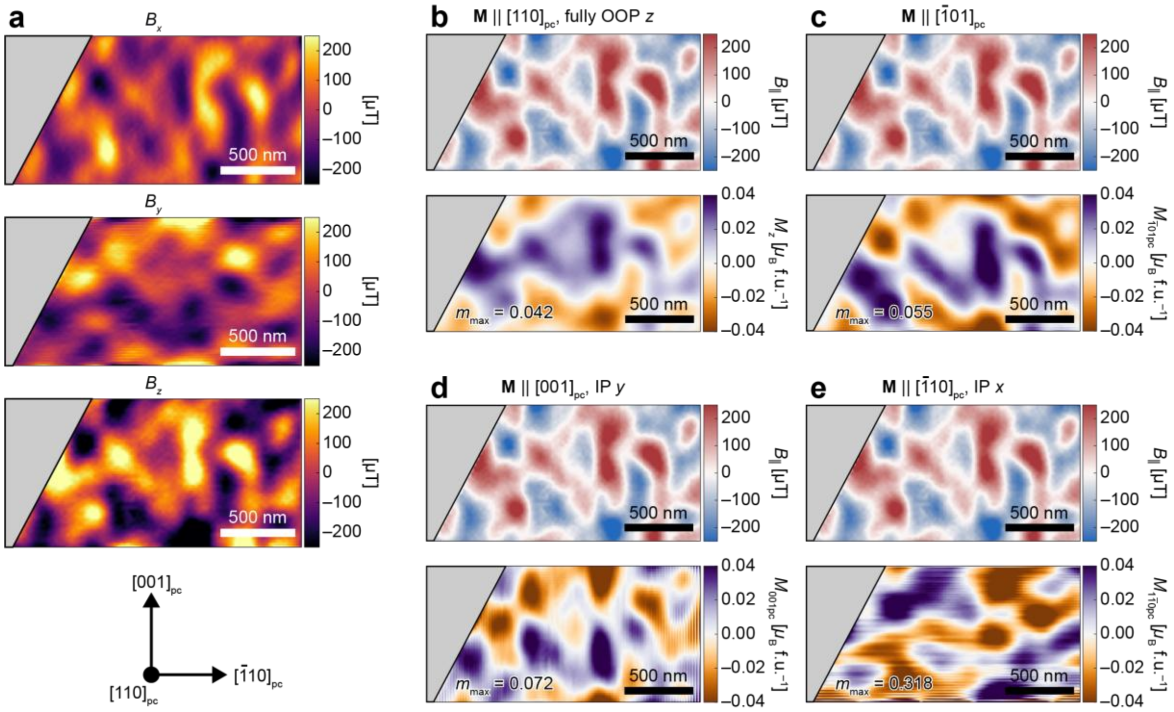

Figure S2 | Reconstruction of magnetization from NV magnetometry. a) Reconstruction of the $B_{x,y,z}$ from $B_{\theta,\phi}$ measured directly with NV magnetometry. b–e) Solutions for M_l from a where l is an assumed uniaxial configuration along (b) $[110]_{pc}$, (c) $[\bar{1}01]_{pc}$, (d) $[001]_{pc}$, or (e) $[\bar{1}10]_{pc}$. The calculated maximum value of the magnetization is shown as an inset. In (b), the value of the magnetization, $0.04\,\mu_B$, matches well with experiment, agreeing with the supposition that the magnetization is primarily in the OOP direction. In (c – e), the values of the magnetization become almost unphysically large.

OOP ferroelectric domains change via IP poling

Comparing OOP PFM phase image in as-grown state in **Figure S3** with other OOP PFM phase images, black areas were reduced. This validates that polarization that was not originally in the electric field direction altered with the electric field direction, and thus, the black region in the OOP PFM phase image indicates OOP polarization.

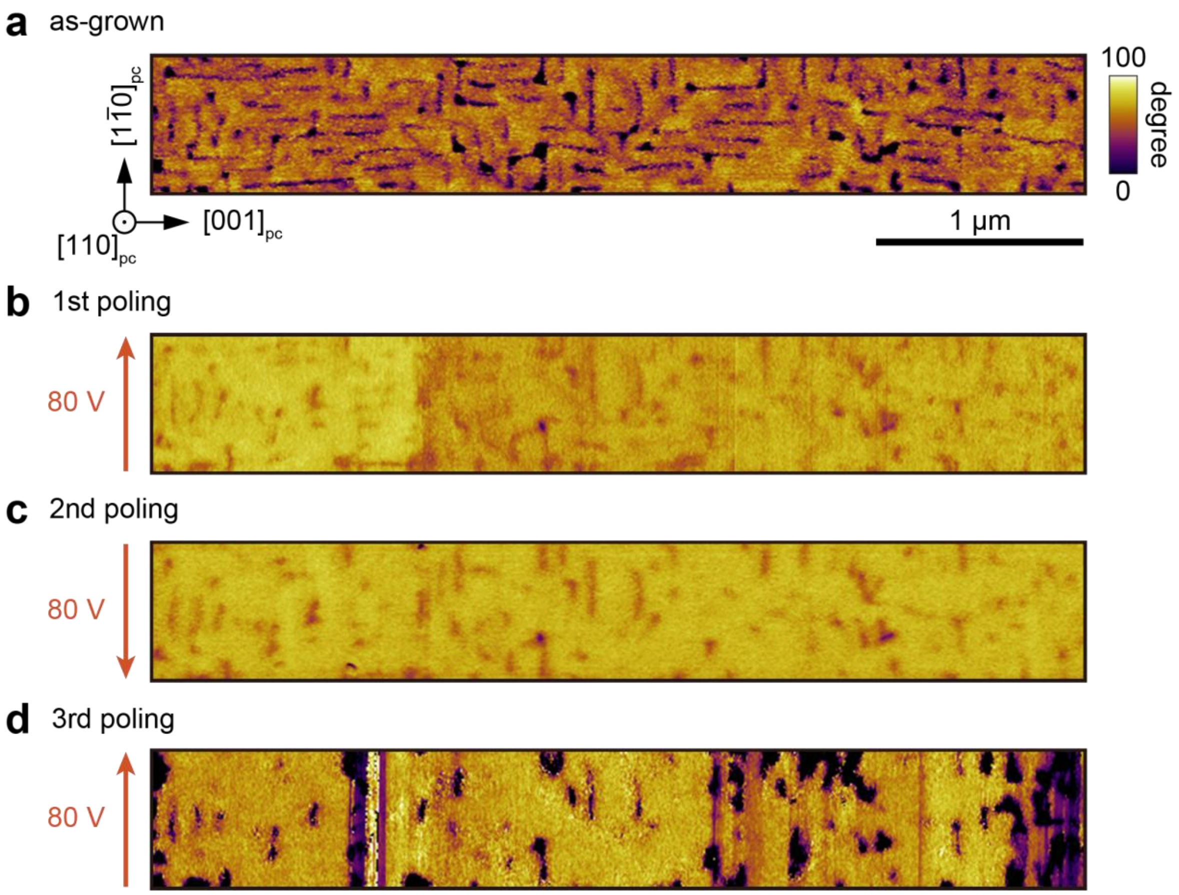

Figure S3 | a–d) The OOP PFM phase images in (a) as-grown state, after (b) first, (c) second, and (d) third polings at 80 V via a Pt coplanar electrode.

IP ferroelectric and magnetic domains change via IP poling

The two IP PFM phase images in as-grown state, after first, second, and third switching events at ±80 V are shown in **Figure S4**a–d, respectively. The arrows on the right-hand side of the IP PFM phase images indicate the detection directions of electric polarizations. The color contrasts of the IP PFM phase images identifying $[001]_{pc}$ component of the polarization (top left) in Figure S4a–d remained unchanged, indicating that only the polarization component parallel to the electric field was reversed, i.e., 109° polarization switching was achieved across the majority of the area.

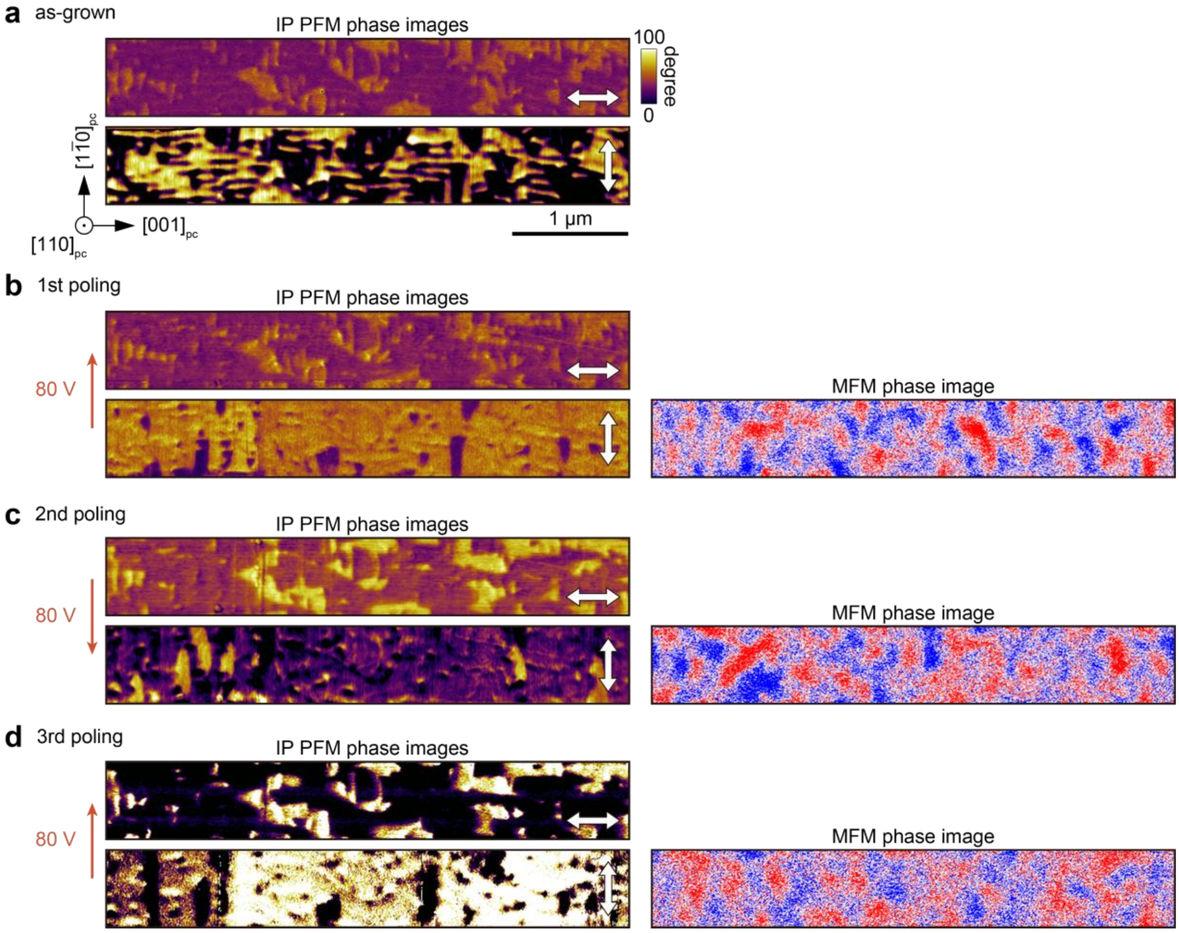

Figure S4 | a–d) The IP PFM phase images and MFM phase images in (a) as-grown state, after (b) first, (c) second, and (d) third polings at ±80 V via a Pt coplanar electrode. IP PFM phase images in the top left (bottom left) identify electric polarization in $[001]_{pc}$ ($[1\bar{1}0]_{pc}$) direction. Arrows on the right-hand side of the IP PFM phase images indicate the detection directions of electric polarizations.

Electric and magnetoelectric switching processes in relaxed thin film of BFCO

As observed in reciprocal space maps around the 310_{pc} reflection, the strain is relaxed along the $[1\bar{1}0]_{\text{pc}}$ axis, while the lattice parameter along the $[001]_{\text{pc}}$ axis remains under ≈0.5% compressive strain. To model this experimentally relaxed structure, we applied a compressive strain of ≈0.5% along the $[001]_{\text{pc}}$ axis while maintaining the lattice parameters along other two directions as in the completely density functional theory-optimized structure. Based on these lattice parameters, we relaxed the atomic positions. Subsequently, we estimated the lowest energy path associated with the 109° switching of electric polarization due to the application of an electric field along the $[1\bar{1}0]_{\text{pc}}$ and the corresponding magnetoelectric switching processes. A key observation is that the qualitative nature of the magnetoelectric switching mechanism remains unchanged compared to the case under 0.5% biaxial compressive strain. Detailed analysis revealed the following: (1) the reversal of electric polarization occurs via the $Ima2$ ($a_0^- a_0^- c_+^0$) state, which involves surmounting a relatively lower energy barrier of ≈269 eV compared to the case under 0.5% biaxial compressive strain (as shown in **Figure S5**). In fact, in the specific $(110)_{\text{pc}}$-oriented thin film geometry, where we confined our investigation of electric polarization switching through IP components of $\mathbf{P}$, as the thin film geometry is less likely to allow switching through the OOP components of $\mathbf{P}$, we find the qualitative conclusion remains the same even under biaxial tensile strain, as denoted in Figure S5. (2) The antiferromagnetic order parameter ($\mathbf{L}$) tends to lie in the $(110)_{\text{pc}}$ plane with an OOP component of magnetization along the $[110]_{\text{pc}}$ axis, i.e., $\mathbf{L} \perp [110]_{\text{pc}}$ and $\mathbf{M} \parallel [110]_{\text{pc}}$, similar to the case under 0.5% biaxial compressive strain (as illustrated in **Figure S6**). (3) During the switching of electric polarization ($\mathbf{P} \parallel [1\bar{1}1]_{\text{pc}} \rightarrow \mathbf{P} \parallel [\bar{1}11]_{\text{pc}}$), $\mathbf{L}$ rotates clockwise, leading to the reversal of the magnetization, as illustrated in **Figure S7**. Therefore, we conclude that structural relaxation does not introduce any qualitative differences in the 109° switching of electric polarization and magnetoelectric process compared to the theoretical predictions made by studying the system under 0.5% biaxial compressive strain.

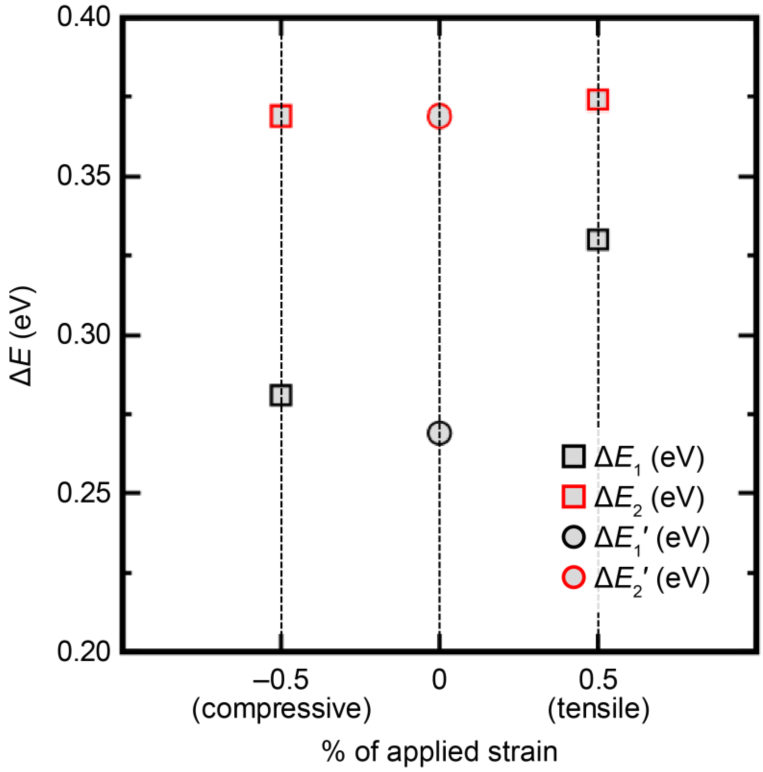

Figure S5 | The estimated energy barriers associated with the ferroelectric 109° switching paths that reverse the polarization direction along the $[1\bar{1}0]_{\mathrm{pc}}$ crystallographic axis (i.e., $[P,-P,P] \rightarrow [-P,P,P]$) are illustrated. These barriers were calculated using nudged elastic band simulations under the application of ≈0.5% biaxial compressive and tensile strain. The energy barriers associated with the $Ima2$ $(a_0^- a_0^- c_+^0)$ and $Iba2$ $(a_0^0 a_0^0 c_+^-)$ intermediate states are denoted as ΔE_1 and ΔE_2, respectively. The results corresponding to the relaxed thin film geometry are represented by circles ($\Delta E_1{}'$ and $\Delta E_2{}'$).

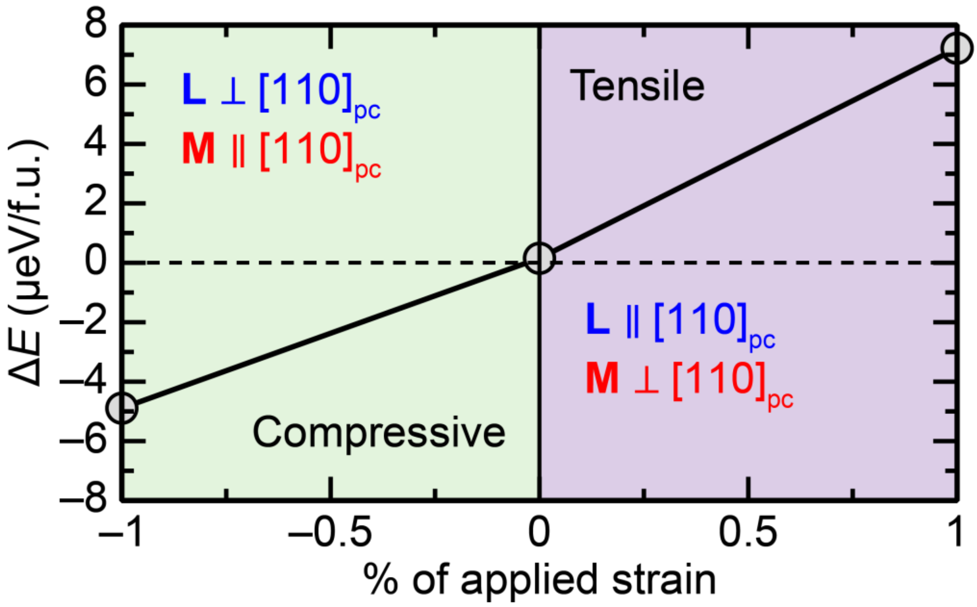

Figure S6 | The calculated energy difference between the IP-oriented antiferromagnetic state and its OOP counterparts ($\Delta E = E[\mathbf{L} \perp [110]_{\mathrm{pc}}] - E[\mathbf{L} \parallel [110]_{\mathrm{pc}}]$) are illustrated, considering the effects of compressive and tensile strain.

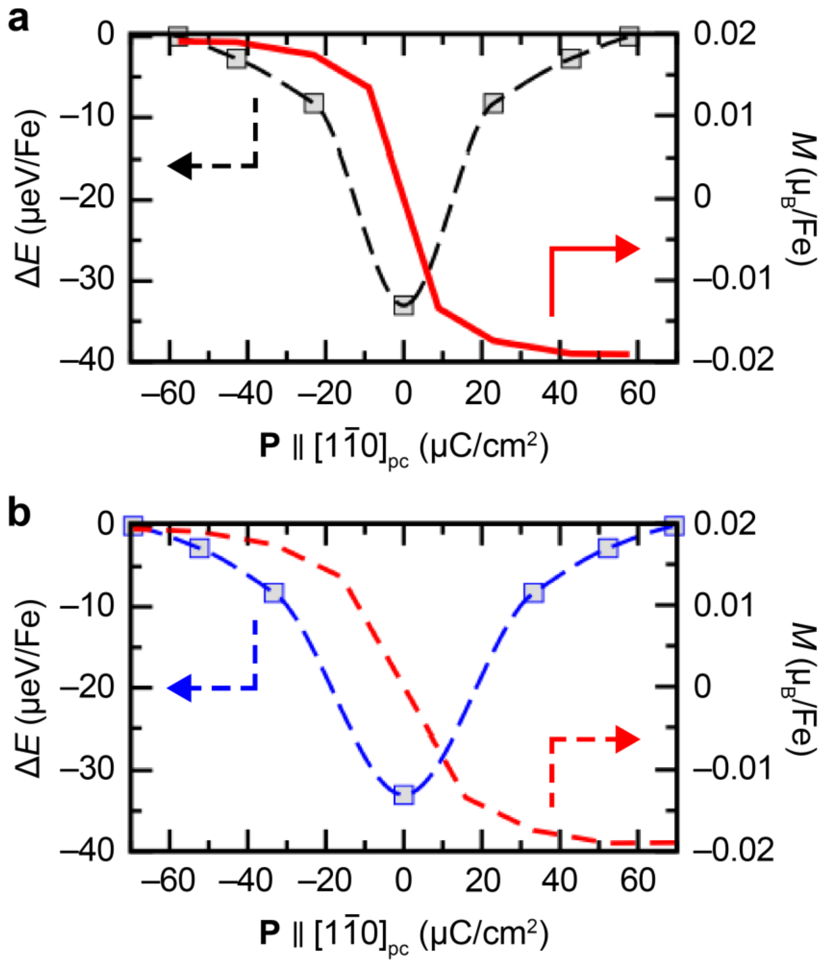

Figure S7 | In the case of a 109° switching, the $Q_{\Gamma_4^-}$ order parameter rotates clockwise around $[110]_{\mathrm{pc}}$. a,b) The estimated relative energy difference between the clockwise rotation of the G-type antiferromagnetic order parameter $\mathbf{L}$ and the anti-clockwise rotation is presented for (a) the system under 0.5% biaxial compressive strain and (b) the model representing the experimentally relaxed structure, respectively.

Analysis about incomplete polarization switching regions

To obtain further insights about incomplete switching in Figure 6d, we visualized how many switching events occurred in the region of Figure 6a, as shown in **Figure S8**. It was found that the zero switching areas were vertically elongated perpendicular to the interface of Pt-electrode/BFCO. The region that underwent two 109° switchings accounted for 48%, while the one and zero 109° switchings were both 26%. These characteristic domains are considered to associate with incomplete domain switching. This speculation is supported by the PFM result after the poling 20 times (**Figure S9**). We observe an increase in domain width after the poling cycles, consistent with the trend in our previous study.[4] In addition, appearance of perpendicularly elongated domains to the interface of Pt-electrode/BFCO is observed (shown by red rectangles). A similar vertically elongated domain was also observed in the OOP PFM image after the third poling in Figure S3.

The formation of such domains in a planar capacitor configuration has been previously reported with $BiFeO_3$ films by Zou et al.[5] They revealed that these domains are related to the polarization fatigue and originate from the growth of pinning domains across the electrodes, which was attributed to the interfacial charge accumulation due to injected electrons from the electrode and the distribution of oxygen vacancies. This effect is more pronounced in materials exhibiting higher leakage currents.

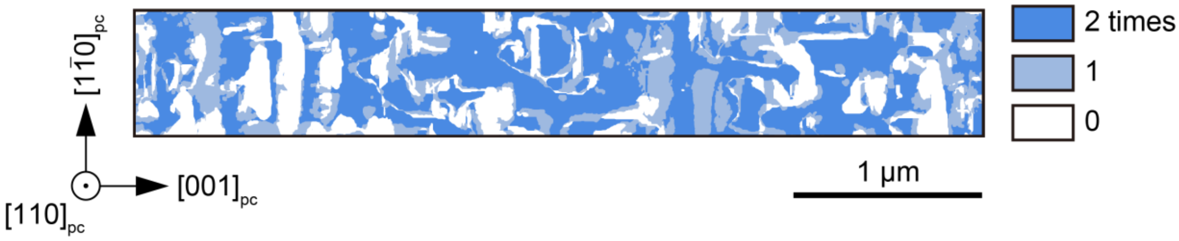

Figure S8 | Map of the number of 109° switching events derived from the 3D-PFM image in Figure 6a.

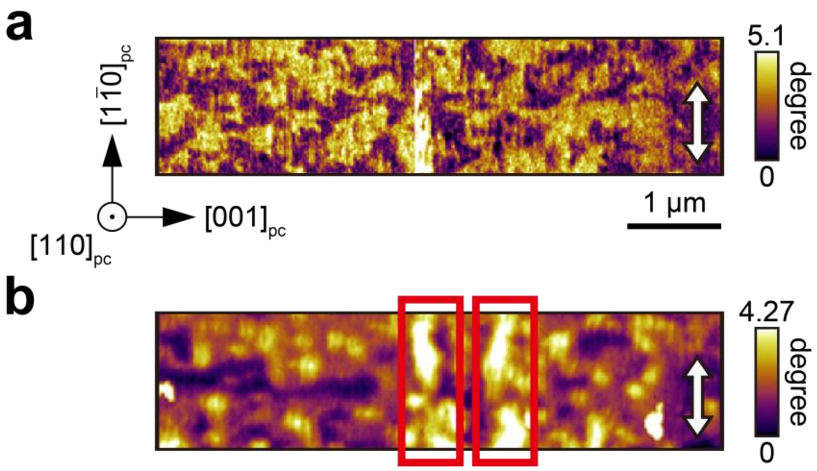

Figure S9 | a,b) IP PFM phase images in (a) as-grown state and (b) after poling 20 times. The arrows at the right-hand side indicate the detection directions of the electric polarizations.

Supp. References

1. Broadway, D. A. et al. Improved Current Density and Magnetization Reconstruction Through Vector Magnetic Field Measurements. *Phys. Rev. Appl.* **14**, 024076 (2020).
2. Dovzhenko, Y. et al. Magnetostatic twists in room-temperature skyrmions explored by nitrogen-vacancy center spin texture reconstruction. *Nat. Commun.* **9**, 2712 (2018).
3. Zhong, H. et al. Quantitative Imaging of Exotic Antiferromagnetic Spin Cycloids in $BiFeO_3$ Thin Films. *Phys. Rev. Appl.* **17**, 044051 (2022).
4. Shigematsu, K. et al. Magnetic Domain Change Induced by In-Plane Electric Polarization Switching in $Bi(Fe, Co)O_3$ Thin Film. *Adv. Phys. Res.* **2**, 2200099 (2023).
5. Zou, X. et al. Mechanism of Polarization Fatigue in $BiFeO_3$. *ACS Nano* **6**, 8997 (2012).
